# Lunch patterns of Brazilian adults and their association with socioeconomic and demographic characteristics: the 2017-2018 *Brazilian National Dietary Survey*

**DOI:** 10.1590/0102-311XEN071724

**Published:** 2025-04-11

**Authors:** Gustavo Henrique Rovari, Ana Clara do Patrocínio Rezende, Rafaella dos Santos Chaves Andreão, Silvia Nazaré Braga Pereira, Ana Lorena Lima Ferreira, Bruna Kulik Hassan, Valéria Troncoso Baltar

**Affiliations:** 1 Instituto de Saúde Coletiva, Universidade Federal Fluminense, Niterói, Brasil.; 2 Universidade Federal do Rio de Janeiro, Rio de Janeiro, Brasil.; 3 ACT Promoção da Saúde, Rio de Janeiro, Brasil.

**Keywords:** Diet, Dietary Patterns, Lunch, Adult, Dieta, Padrões Dietéticos, Almoço, Adulto, Dieta, Patrones Dietéticos, Almuerzo, Adulto

## Abstract

Lunch is an important meal in Brazil, with varying standards among different population groups. This study aims to determine the lunch dietary patterns of Brazilian adults and verify their association with socioeconomic and demographic characteristics. Data from the *Brazilian National Dietary Survey* were used, comprising 28,901 adults aged 19 to 59 years (excluding pregnant and lactating women). Food consumption was assessed via 24-hour recalls at the interviewee’s home. A total of 1,832 food items were recorded, 1,120 of which were mentioned at lunch. Socioeconomic and demographic variables were obtained through a structured questionnaire. Dietary patterns were derived by factor analysis, considering the complexity of the sample. The mean scores and 95% confidence intervals for each dietary pattern were estimated across all levels of socioeconomic and demographic variables. Considering the complexity of the sampling design, all analyses were performed using the statistical package SAS OnDemand for Academics. In total, 3.4% of Brazilian adults reported not having lunch. In total, three patterns explained 30.7% of lunch variability: traditional Brazilian; salads; and protein-based meal with desserts and beverages. Men, individuals with less schooling, those with lower income, and residents of the Central-West and Northeast regions adhered more to the traditional pattern. Fruits did not play an essential role in any pattern. Adherence to dietary patterns varied according to socioeconomic and demographic factors.

## Abstract (pt)

O almoço é uma refeição importante no Brasil cujo padrão varia entre os seus grupos populacionais. Este estudo buscou determinar os padrões alimentares de adultos brasileiros durante o almoço e verificar sua associação com as características socioeconômicas e demográficas dessa população. Os dados da *Pesquisa Nacional de Alimentação* (que incluiu 28.901 adultos com idade entre 19 a 59 anos) foram utilizados (excluindo-se gestantes e lactantes). O consumo alimentar foi avaliado por recordatórios de 24 horas nos domicílios dos entrevistados. Foram registrados 1.832 alimentos, dos quais 1.120 foram consumidos no almoço. As variáveis socioeconômicas e demográficas foram obtidas por um questionário estruturado. Os padrões alimentares foram derivados por análise fatorial considerando-se a complexidade da amostra. A média e os intervalos de 95% de confiança dos escores para cada padrão alimentar foram estimados em todos os níveis de variáveis socioeconômicas e demográficas. Considerando a complexidade do desenho amostral, todas as análises foram realizadas pelo pacote estatístico SAS OnDemand for Academics. No total, 3,4% dos adultos brasileiros não reportaram almoçar. Três padrões explicaram 30,7% da variabilidade do almoço: tradicional brasileiro; saladas; e à base de proteínas com sobremesas e bebidas. Homens, indivíduos com menor escolaridade e menor renda e os residentes no Centro-oeste e Nordeste aderiram mais ao padrão tradicional brasileiro. As frutas não desempenharam um papel essencial em nenhum padrão. A adesão aos padrões alimentares varia de acordo com fatores socioeconômicos e demográficos.

## Abstract (es)

El almuerzo es una comida importante en Brasil cuyo patrón varía entre los grupos poblacionales. Este estudio buscó determinar los patrones dietéticos de los adultos brasileños durante el almuerzo y verificar su asociación con las características socioeconómicas y demográficas de esta población. Se utilizaron datos de la *Encuesta Nacional de Alimentación* (que incluyó a 28.901 adultos de entre 19 y 59 años) -excluyendo a las mujeres embarazadas y lactantes. El consumo de alimentos se evaluó mediante recordatório alimentário de 24 horas en los hogares de los entrevistados. Se registraron 1.832 alimentos, de los cuales 1.120 se consumieron en el almuerzo. Las variables socioeconómicas y demográficas se obtuvieron mediante un cuestionario estructurado. Los patrones dietéticos se derivaron mediante análisis factorial teniendo en cuenta la complejidad de la muestra. La media y los intervalos de 95% de confianza de las puntuaciones para cada patrón dietético se estimaron en todos los niveles de variables socioeconómicas y demográficas. Teniendo en cuenta la complejidad del diseño de la muestra, todos los análisis se realizaron utilizando el paquete estadístico SAS OnDemand for Academics. En total, el 3,4% de los adultos brasileños declararon que no almorzaban. Además, tres patrones explicaron el 30,7% de la variabilidad del almuerzo: tradicional de Brasil; ensaladas; y a base de proteínas con postres y bebidas. Los hombres, las personas con menor nivel de estudios, con menores ingresos y los residentes en las regiones Centro-Oeste y Nordeste registraron más el patrón tradicional de Brasil. Las frutas no jugaron un papel esencial en ningún patrón. La adherencia a los patrones dietéticos varía según los factores socioeconómicos y demográficos.

## Introduction

Lunch is one of the most consumed meals in Brazil, crucial to daily energy contribution and represents the Brazilian’s food culture ^1^ ^,^ ^2^. It is essential for maintaining healthy food habits, as rice and beans, both traditional staple foods of the Brazilian diet, are its most prevalent components and are essential sources of energy, protein, vitamins, and minerals ^2^ ^,^ ^3^. However, changes in lifestyle of the Brazilian population have directly impacted food consumption over the past few decades, resulting in increased intake of ultra-processed products and decreased consumption of rice and beans ^4^ ^,^ ^5^ ^,^ ^6^. Despite this, little is known about the specific patterns that characterize lunch among Brazilians.

Traditionally, studies that assess food consumption have focused on the intake of macro and micronutrients, often disregarding aspects that involve the combination of foods consumed in a meal. Usually, consumption is not of just one food item but a set of them, facilitating the assessment of diet complexity ^7^ ^,^ ^8^. Therefore, studying lunch from a consumption pattern perspective is highly relevant, especially considering that lunch is the meal with the highest energy contribution in Brazil. As one of the three main meals, elucidating its composition is necessary to improve dietary guidance.

The *Dietary Guidelines for the Brazilian Population* ^3^ recommend that meals prioritize unprocessed and minimally processed foods to promote an adequate and healthy diet. However, adhering to these recommendations is challenging, as the supply of unhealthy ultra-processed products is expanding at increasingly lower prices. Moreover, other issues also hinder the production and consumption of regular meals, such as long working hours - which limit the time dedicated to meal preparation - and limited access to and higher price of healthy foods ^9^. Socioeconomic barriers influence not only the number of daily meals but also their quality ^2^. Several sociodemographic and economic factors, such as household income and schooling level, have been associated with the consumption of more affordable foods that show lower quality, higher energy density, and rich in fat and sugar ^10^ ^,^ ^11^ ^,^ ^12^.

Thus, this study aims to analyze the lunch dietary patterns of Brazilian adults and examine their association with socioeconomic and demographic characteristics, using data from the *Brazilian National Dietary Survey* (INA, acronym in Portuguese) conducted in a subsample of the 2017-2018 *Brazilian National Household Budget Survey* (POF, acronym in Portuguese) ^4^.

## Materials and methods

### Study population and definition of food groups

Data were obtained from the INA and the 2017-2018 POF. The INA contains food consumption information for 20,112 households randomly selected from the POF (representing 34.7% of the total households), encompassing data for 46,164 residents (all aged ≥ 10 years). In the 2017-2018 INA, food consumption was assessed using two 24-hour recalls (24hR) conducted at the respondent’s home on non-consecutive days. Moreover, during data collection, participants answered the occasion of consumption (breakfast, lunch, afternoon snacks, dinner, supper, or other occasions).

A data entry program was adopted in the interviews, and each food item included information on its unit of measurement, quantity, and occasion of consumption. Lunch was defined as all items consumed during the lunch occasion. A total of 1,832 food items were recorded, of which 1,239 were mentioned at lunch and 1,120 were reported in the first 24hR. These items were initially sorted into 28 groups based on their nutritional characteristics or food type. Some groups were disregarded for various reasons, including low consumption, lack of characterization as a defined food group, or because they reflect lifestyle rather than just food consumption (water, supplements, sauces, condiments, mixed preparations, and alcoholic beverages).

Socioeconomic and demographic variables were obtained from the POF, and such data were answered by the household’s reference person, containing information on age, sex, household income, schooling, and region of residence.

Food items consumed at lunch by adult residents aged 19-59 were selected, as reported in the first 24hR for this study. In the final sample, individuals of both sexes were included, excluding pregnant and lactating women, totaling 28,901 individuals (of whom 710 did not consume any food item identified as lunch).

It is worthy to mention that this study was conducted based on a secondary database from the Brazilian Institute of Geography and Statistics (IBGE, acronym in Portuguese). Therefore, approval from a research ethics committee was not required, as provided by *Resolution n. 510/2016* of the Brazilian National Health Council.

### Statistical analysis

A first pool of 28 food groups was evaluated. Some groups were excluded due to low consumption at lunch or low communality in the factor analysis (tea, coffee, dairy products, oilseeds, baked goods, bread, pizzas, snacks and sandwiches, soups and broths, breakfast cereals, other cereals, viscera, and other meats). After exclusion and regrouping, 14 food groups remained (based on consumption from the first 24hR, in grams - Box 1). Dietary patterns were obtained by factor analysis, employing principal component extraction followed by varimax rotation ^13^. Factor analysis was initially performed using a correlation matrix, estimated considering sample complexity across the 28 groups ^14^.

**Box 1:** Grouping of lunch food items from the 2017-2018 *Brazilian National Dietary Survey*.

FOOD GROUPS	FOOD ITEMS
Rice	Varieties of rice, brown rice, and rice-based preparations
Beans	Variety of beans, bean-based preparations, meat substitute legumes, and other legumes
Leafy greens	Lettuce, kale, cabbage, raw salad, and other leafy greens
Vegetables	Pumpkin, carrot, chayote, cucumber, tomato, and other vegetables
Roots and tubers	Sweet potato, English potato, cassava, and other tubers
Fruits	Pineapple, *açaí*, banana, orange, apple, papaya, mango, watermelon, tangerine, grape, dried fruits, and other fruits
Flours and pasta	Cassava flour, *farofa*, pasta, instant noodles, macaroni, and macaroni-based preparations
White meat	Poultry, poultry-based preparations, fish, and fish-based preparations
Eggs	Eggs and egg-based preparations (tortilla, omelet with meat, vegetables, cheese, ham, and egg Benedict)
Oil and fat	Butter with or without salt, bacon, lard, coconut milk, soy, corn, coconut oil, crackling, and olive oil
Drinks	Water, juices, refreshments, dairy drinks, and soy-based drinks
Soft drinks	Diet soft drinks and soft drinks
Sweets	Chocolates, chocolate milk, milk-based sweets, peanut-based sweets, fruit-based sweets, ice cream/popsicles, honey/raw sugar and table sugar, sweeteners, and other sweets
Red/Processed meats	Beef, beef preparations, pork, salted meat, processed meats, cold cuts and sausages, and other types of meat

For factor selection and naming, the food groups with factor loadings greater than 0.30, in module, were considered. This naming was based on interpretability, the characteristics of the groups retained in each pattern, and terminologies recognized in previous studies on dietary patterns ^5^.

Finally, factor scores were computed employing the regression method, representing estimates of each individual’s adherence to the factor analysis ^13^. Mean scores of lunch patterns and their respective 95% confidence intervals (95%CI) were estimated for each category of variables, including sex (male, female), age group (19-39 years, 40-59 years), region (North, Northeast, Southeast, South, Central-West), schooling (< 9 years of study, 9 or more years of study), and per capita income. The latter considered total monthly income divided by the number of residents in the consumption unit, classified in minimum wages effective on January 15, 2018 (up to 0.5 minimum wages, between 0.5 and 1 minimum wage, between 1 and 2 minimum wages, greater than 2 minimum wages).

Considering the sample design complexity, all analyses were performed using the SAS OnDemand for Academics statistical package (https://www.sas.com/).

## Results

According to the 2017-2018 INA data, only 3.37% of Brazilian adults reported not consuming any lunch-related food items. Approximately 50.15% of the adults who reported having lunch were female, 52.93% were aged 19-39 years, and 42.4% lived in the Southeast. The most significant proportion of adults had a per capita household income greater than 2 minimum wages (37.53%), and 69.56% had nine or more years of schooling.

The adult food consumption data showed three dietary patterns of lunch, accounting for almost 31% of the total variance, with each pattern defined by the foods/food groups showing the highest and lowest factor loadings (Table 1). The first pattern corresponds to the “traditional Brazilian” lunch, in which there was higher consumption of rice, beans, red/processed meats, and beverages, and lower consumption of flour, pasta, and soft beverages. The second pattern, named the “salads” pattern, was characterized by higher consumption of leafy greens, vegetables, oils, and fats and lower consumption of sweets. Finally, the third pattern, named the “protein-based meal with desserts and beverages” pattern, was characterized by higher consumption of white meat, sweets, and beverages, along with lower relative consumption of soft beverages and red/processed meats. We found that “fruits” did not show enough loadings to characterize any of the three patterns.

**Table 1:** Factor loadings of dietary patterns obtained via factor analysis of lunch food consumption data of Brazilian adults. 2017-2018 *Brazilian National Dietary Survey*.

Food groups	Traditional Brazilian staple	Salads	Protein-based meal with desserts and beverages	Communality
Rice	0.73	-0.08	0.14	0.56
Beans	0.72	-0.08	0.10	0.53
Leafy greens	-0.04	0.66	0.15	0.45
Vegetables	0.06	0.41	-0.02	0.17
Roots and tubers	-0.01	0.18	-0.05	0.04
Fruits	-0.11	0.01	-0.05	0.01
Flours and pasta	-0.41	-0.16	0.05	0.20
White meat	-0.18	-0.31	0.42	0.30
Eggs	0.06	0.09	-0.03	0.01
Oils and fats	-0.11	0.78	0.15	0.64
Drinks	0.17	0.04	0.72	0.54
Soft drinks	-0.11	-0.02	-0.28	0.09
Sweets	0.01	0.05	0.62	0.39
Red/Processed meats	0.39	0.26	-0.36	0.35
Eigenvalue	1.47	1.45	1.37	-
Variance explained for each factor	10.53%	10.38%	9.76%	-
Accumulated explained variance	10.53%	20.91%	30.67%	-

The “traditional Brazilian” lunch pattern was associated with being a man, whereas the “salads” pattern was associated with being a woman. No significant differences were found between sexes regarding the “protein-based meal with desserts and beverages” pattern. Adherence to the “salads” pattern was the only pattern that differed between age groups, with greater consumption among older adults than young adults (mean score of 4.79 [95%CI: 2.15; 7.42] versus -4.26 [95%CI: -6.95; -1.56], respectively). The Central-West and Northeast Brazilian regions stood out for the “traditional Brazilian” lunch pattern, whereas the South, Central-West, and Southeast regions showed a greater tendency for the “salads” pattern. The “protein-based meal with desserts and beverages” pattern prevailed in the Northeast (Table 2).

**Table 2:** Factor scores by socioeconomic and demographic variables. 2017-2018 *Brazilian National Dietary Survey*.

Parameter	Traditional Brazilian staple	Salads	Protein-based with deserts and drinks
Mean (95%CI)	Mean (95%CI)	Mean (95%CI)
Sex			
Male	16.98 (14.15; 19.81)	-4.24 (-7.10; -1.38)	1.70 (-1.13; 4.53)
Female	-16.88 (-18.77; -14.99)	4.21 (2.02; 6.41)	-1.69 (-3.81; 0.43)
Age group (years)			
19-39	1.78 (-0.83; 4.38)	-4.26 (-6.95; -1.56)	1.44 (-1.26; 4.13)
40-59	-2.00 (-4.52; 0.52)	4.79 (2.15; 7.42)	-1.62 (-4.10; 0.87)
Region			
North	-13.77 (-19.14; -8.39)	-26.28 (-30.51; -22.05)	-8.95 (-14.34; -3.57)
Northeast	7.37 (4.35; 10.38)	-24.94 (-26.96; -22.91)	13.62 (10.66; 16.57)
Southeast	-1.05 (-4.54; 2.43)	8.80 (5.31; 12.28)	-4.37 (-8.03; -0.72)
South	-17.08 (-22.25; -11.90)	23.03 (15.21; 30.86)	1.96 (-3.14; 7.07)
Central-West	26.64 (20.01; 33.26)	21.97 (15.67; 28.27)	-16.63 (-21.14; -12.12)
Schooling (years of study)			
< 9	12.41 (9.68; 15.14)	-12.48 (-15.08; -9.88)	-1.11 (-3.86; 1.65)
9 or more	-5.43 (-7.82; -3.04)	5.46 (2.85; 8.07)	0.48 (-1.97; 2.94)
Per capita income (minimum wages)			
Up to 0.5	11.54 (6.51; 16.58)	-28.38 (-32.21; -24.56)	-1.93 (-6.69; 2.82)
Between 0.5 and 1	9.35 (4.82; 13.89)	-17.93 (-21.10; -14.75)	2.86 (-1.60; 7.32)
Between 1 and 2	4.76 (1.75; 7.77)	-1.84 (-4.87; 1.19)	-2.63 (-6.00; 0.74)
Greater than 2	-12.19 (-15.54; -8.83)	18.71 (14.56; 22.86)	1.23 (-2.34; 4.80)

95%CI: 95% confidence interval.

Note: bold values indicate a statistically significant direct association.

Regarding schooling, when comparing adults with < 9 years of studing and with 9 or more years of study, we observed that the former showed greater adherence to the “traditional Brazilian” pattern and the latter greater adherence to the “salads” pattern. Finally, greater adherence to the traditional Brazilian lunch was observed among adults with incomes of up to 2 minimum wages compared to those with incomes greater than 2 minimum wages. In contrast, adults with incomes above 2 minimum wages predominantly adhered to the “salads” pattern, more than all other income ranges. The adherence to “protein-based meal with desserts and beverages” pattern was similar for all income groups.

## Discussion

In this study, three dietary patterns were derived from the lunch consumption data of Brazilian adults: the “traditional Brazilian” pattern, the “salads” pattern, and the “protein-based meal with desserts and beverages” pattern. The first pattern, which remained the same after rotation, is characterized by a greater consumption of rice, beans, red/processed meats, and beverages - a pattern that has been found as the primary pattern in other Brazilian studies ^5^. Higher adherence to this pattern was observed among men, Central-West residents, individuals with < 9 years of study, and those with a per capita income of up to 2 minimum wages. The “salads” pattern, characterized by higher consumption of leafy greens, vegetables, oils, and fats (including olive oil), showed greater adherence among women, adults aged 40-59, Central-West, South, and Southeast residents, with greater schooling levels (9 or more years of study), and those with a per capita income greater than 2 minimum wages. Finally, the “protein-based meal with desserts and beverages” pattern, characterized by the highest consumption of white meat, sweets, and beverages (excluding soft beverages), differed only by region, with a predominant adherence among individuals from the Northeast.

Almost the entire Brazilian population eats lunch (96.63%), a frequency higher than that for breakfast and dinner (92.59% and 87.2%, respectively; data not shown). These findings lead to the understanding that lunch is the main meal in Brazil and confirms the crucial role of lunch in the daily lives of Brazilians ^2^.

The significant adherence to the “traditional Brazilian” pattern corroborates other studies that highlighted that rice and bean consumption remains among Brazilians’ usual food habits, along with meat/processed meat consumption ^5^ ^,^ ^15^ ^,^ ^16^. In this sense, we can affirm that the classic Brazilian lunch pattern is still frequent ^17^, which is a positive indicator of nutritional quality for the main meal of the day for Brazilian adults, especially in the economically active age group, mainly, younger adults. This traditional combination of foods including rice and beans is described in the literature as an important healthy eating marker and a protective factor against noncommunicable diseases (NCDs), such as cardiometabolic disorders ^18^. However, a recent nationally representative study comparing Brazilians overall food consumption patterns over a 10-year period (2008-2009 and 2017-2018) found that the traditional staple has been losing ground over time ^6^. Although studies showed a lower consumption of rice and beans, lunch with the “traditional Brazilian” pattern continues to be an important pattern (the one with the most significant variability).

The adherence to the “traditional Brazilian” pattern was associated with lower schooling and income levels. Previous studies have suggested that low schooling is a determinant of consumption patterns characterized by a higher presence of ultra-processed products ^10^. In Brazil, while people in vulnerable situations with less purchasing power still consume traditional meals (such as rice and beans) and nutritious meals for lunch, they are also increasingly exposed to ultra-processed products due to lower prices and the greater offers. Combined with that, the difficulty to afford a healthy diet, generally at a higher cost, results in a worrisome decreased consumption of rice and beans by Brazilians ^4^ ^,^ ^5^ ^,^ ^6^. 

The advance of ultra-processed food consumption is a public health issue, mainly because it is associated with a lower nutritional quality of food diet - higher in fat, sugar, and salt - and an increased risk of several NCDs, such as obesity, type 2 diabetes mellitus, and hypertension ^19^ ^,^ ^20^ ^,^ ^21^ ^,^ ^22^. Moreover, ultra-processed food availability is based on unsustainable food systems with significant environmental impacts, and it usually shows lower costs compared to unprocessed or minimally processed foods, making them more accessible to the population ^23^. 

In the “salads” pattern, we found greater adherence to a diet rich in vegetables, salads, good fats, and lean proteins. This pattern was mainly adopted by women, older individuals with higher schooling levels, and people from the Central-West, South, and Southeast regions. This pattern was also observed in studies that have evaluated overall food consumption patterns among adults ^15^ ^,^ ^16^ ^,^ ^24^. Although this pattern contains healthy items ^24^, adherence to it does not necessarily indicate healthy food consumption. For instance, a study with university students found that although 45% of the sample considered their diet healthy, only three of the ten healthy eating recommendations contained in the *Dietary Guidelines for the Brazilian Population* ^3^ showed good adherence (≥ 60%) ^25^.

We emphasize that the “salads” pattern partly showed important food components recommended by the *Dietary Guidelines for the Brazilian Population*. It was more commonly adopted by individuals with higher schooling levels (9 or more years of study) and those with higher per capita income, suggesting that these foods are more accessible to the population with higher socioeconomic status and residing in the country’s wealthiest regions, namely, the Central-West, South, and Southeast regions. Regarding the association with the female sex and considering that certain items of this pattern are recommended as healthier by the *Dietary Guidelines for the Brazilian Population*, adult women have shown healthier behavior than adult men regarding healthcare, lifestyle, and food consumption ^26^. However, healthy items such as rice and beans, also recommended by the *Dietary Guidelines for the Brazilian Population*, were more common in the “traditional Brazilian” pattern and predominated among those with lower socioeconomic status. The consumption of healthier foods across different socioeconomic groups can be influenced by social and cultural issues ^26^ ^,^ ^27^.

On the other hand, the third pattern, composed mainly of high protein foods, sweets (that may indicate consumption of desserts), and beverages reveals an interesting finding about the consumption of different items in an integrated way during lunch. This pattern shows a positive adherence to white meats, beverages (other than soda), and sweets. While patterns consisting of beverages and sweets have been described in the literature ^15^ ^,^ ^16^ in a more general consumption pattern, in our study, these items appear along with white meat in the lunch pattern. Moreover, we found this pattern to show greater adherence to fish (characterized as white meat), which is associated with a Northeast region traditional and local production and consumption. Since the 2008-2009 POF, the highest frequencies of fresh fish consumption have been reported for the North and Northeast, which may represent a positive factor in diet quality. Although changes in these food consumption frequencies were observed over the 10 year-period (2008-2009 POF to 2017-2018 POF), the most recent data still indicate the highest consumption frequencies for the North (21% to 16.6%) and Northeast (9.7% to 8.2%) compared to the Southeast (3.4% to 3.3%), South (2.3% to 3.1%), and Central-West (2.3% to 3.9%) ^4^.

Nonetheless, in the “protein-based meal with desserts and beverages” pattern, a greater adherence to sweets was also observed, probably associated with dessert consumption, which conflicts with the DGBP recommendations that advise a small consumption of sweets due to their adverse health impacts resulting from excessive sugar consumption ^3^. According to *Risk and Protective Factors Surveillance System for Chronic Noncomunicable Diseases Through Telephone Interview* (Vigitel, acronym in Portuguese) in 2021, overweight (body mass index - BMI > 25kg/m^2^) was higher among Brazilian men aged over 18 (59.9%, 95%CI: 57.6; 62.2) than women (55%, 95%CI: 53.0; 57.0). However, obesity (BMI ≥ 30kg/m^2^) was similar between women (22.6%, 95%CI: 21.1; 24.0) and men (22%, 95%CI: 20.0; 24.0) ^28^. Thus, the higher consumption of sweets in this pattern generates concerns since foods with excessive sugar are generally more associated with a higher occurrence of NCDs ^29^.

Our study provides essential and robust findings by analyzying the relationship between lunch patterns and the socioeconomic status across Brazilian regions using nationally representative data. As a strength of this article, we highlight the use of the INA/POF, a crucial tool for analyzing and monitoring the eating habits of the Brazilian population, with a representative sample, which includes the definition of a self-reported meal - being a fundamental database for developing and evaluating public health and nutrition policies ^17^ ^,^ ^30^. Lunch remains scarcely explored in the literature, especially on a national level, which is a strong point of our analysis. In this sense, we believe that the patterns found here reflect Brazilian eating habits in their three most common variations, suggesting the social and political echoes of Brazilian food. Historically, lunch in Brazil represents a relevant moment reserved to eat and carries essential cultural, social, and affective aspects ^31^. Another strength of our study is the use of the sample’s complexity in the factor analysis for factor derivation.

The “traditional pattern”, found as the first pattern, similar to other studies ^5^, suggests that Brazil maintains the traditional “rice-and-bean” pair, mainly among populations with lower schooling and income levels, while also being strongly influenced by the hegemonic, globalized food systems 

This study holds some limitations. The INA selected weekdays and weekends to characterize general eating habits of the population, however it does not represent the usual intake. Moreover, factor analysis for deriving dietary patterns is subject to limitations due to involving certain arbitrary decisions - such as grouping food items, choosing the number of factors, and naming them. Also, correlations between the food items are usually low when studying food patterns. Therefore, the first patterns explained the low variability. Thus, our findings are similar to those of other studies using 24hR, and the variability explained by patterns considering only one meal was very similar to patterns derived for daily consumption ^5^. On the other hand, a strength of this study is using a self-reported meal occasion, avoiding the misclassification when using time and energy classifications. Moreover, the identified lunch eating patterns found in this study were, as expected, comparable with overall consumption patterns from other studies.

Regarding the Brazilian eating habits of the most important meal of the day, our findings can be very promising as a guide for future studies that propose to analyze not only aspects of health, socioeconomic conditions, culture and regional habits but also age and gender, as only a small portion of the Brazilian population reported not having lunch daily.

## Conclusions

To our knowledge, this is the first nationwide representative study that derived lunch patterns among Brazilian adults. This study shows that Brazilian adults have a high prevalence of lunch consumption, reaffirming that it is an important meal of the day. Among those who had lunch, we found three main patterns, and the one with the most significant variability was the “traditional Brazilian” pattern, which includes rice and beans along with a combination of traditional and healthy food habits. This pattern was associated with being male and having a lower income and schooling level. These findings emphasize that the most vulnerable population still maintains traditional and healthier habits, which can provide strategic information for public health decisions.
